# Declining incidence of malaria imported into the UK from West Africa

**DOI:** 10.1186/1475-2875-7-235

**Published:** 2008-11-10

**Authors:** Ron H Behrens, Bernadette Carroll, Valerie Smith, Neal Alexander

**Affiliations:** 1 Department of Travel Medicine, Hospital for Tropical Diseases, Capper St, London, WC1 6JB, UK; 2 HPA Malaria Reference Laboratory, London School of Hygiene and Tropical Medicine, Keppel Street, London, WC1E 7HT, UK; 3 Clinical Research Unit (RHB) and Infectious Diseases Epidemiology Unit (NA) London School of Hygiene and Tropical Medicine, Keppel Street, London, WC1E 7HT, UK

## Abstract

**Background:**

Two thirds of all falciparum malaria cases reported in the United Kingdom (UK) are acquired in West Africa (WA). To ensure recommendations and guidelines for malaria prophylaxis in travellers to West Africa correlate to the risk of infection, a study was undertaken to examine recent trends and predict future patterns of imported malaria acquired by UK residents visiting West Africa and West African visitors to the UK between 1993 and 2006.

**Methods and Results:**

Using passenger numbers and malaria surveillance reports, the data revealed a 2.3-fold increase in travel to West Africa with a five-fold increase in travelers visiting friends and relatives (VFR). Malaria incidence fell through the study period, the greatest decline noted in VFR with a fall from 196 cases/1,000 person-years to 52 cases/1,000 person-years, 9.8% per year p < 0.0001. The risk for travellers from the UK visiting for other reasons declined 2.7 fold, at an annual decrease of 7.0%, with the incidence in West African visitors to the UK falling by 2.3 fold, a rate of 7.9% annually.

**Discussion:**

The reduction in incidence among all three groups of travellers may be explained by several factors; changing chemoprophylaxis usage and/or increased travel in urban areas where malaria risk has declined over the past decade, or widespread reduction in malaria transmission in West Africa.

**Conclusion:**

With the reduction in malaria incidence seen in both visitors to and from West Africa, the most rational explanation for these findings is a fall in malaria transmission in West Africa, which may require a change in chemoprophylaxis policy for UK travelers over the next 5–10 years.

## Background

Malaria remains a threat to travellers visiting endemic regions. Since 1987, approximately 39,000 cases of malaria have been reported to the UK reference laboratory [1]. A number of risk factors for acquiring malaria during travel have been identified, of which destination is the most important. West Africa accounts for approximately two thirds of all cases reported in the UK, with travellers to Nigeria and Ghana making up half of all imported *Plasmodium falciparum *infections [2,1]. The reason for travel is another significant contributory factor and three quarters of all reported cases occur in travellers who have been visiting friends and relatives (VFR)[3,1,4] in West Africa. Failure to take or comply with the correct chemoprophylactic regimens is associated with higher rates of malaria. Unsurprisingly, only 42% of malaria cases report the use of any prophylaxis, and under 10% of VFR cases have used a correct prophylactic regimen [1]. Duration of visit may also be important, with longer exposure presumably increasing the risk of malaria [5].

The current study was designed to inform and estimate the impact of the UK's malaria prevention policy by estimating the changing trend of malaria in travellers to West Africa, and the contribution of known risk factors to morbidity. Malaria incidence in travellers to and from the region has not been examined since 1987 [4].

## Methods

### Data collection

#### Travel by UK residents to malaria endemic countries

Data on overseas travel by UK residents were obtained from the Office for National Statistics (ONS), collected as part of the International Passenger Survey (IPS). The IPS is a year round survey of incoming and outgoing passengers at all major ports as part of the government's data collection for its balance of payments account. Around 250,000 face-to-face interviews of a randomly selected sample of passengers (representing 0.2% of all travellers) provide estimates of the total annual overseas visits made by UK residents, and the number of incoming visits to the UK from other countries. The survey design and passenger flows are used to calculate weights and hence sampling errors [6]. The survey also collects information on the reason for and duration of travel. The units are numbers of visits made, rather than individuals travelling, many of whom may make repeated trips. The study countries were Sierra Leone, Nigeria, Ghana and The Gambia, since these are the most popular destinations in the region for UK residents. For other West African countries, the sample size was too small to assess the denominator accurately. The data were stratified by reason for travel into two major groups: UK residents travelling to visit friends and relatives (VFR) and UK residents travelling for all other purposes such as business, vacation, study and the military (non-VFR). The number of residents from the study region arriving in the UK (overseas visitors) were collected through the same survey and used to estimate the incidence of malaria occurring in visitors to the UK.

#### Surveillance data on malaria

Malaria surveillance reports were provided by the Malaria Reference Laboratory (MRL) of the Health Protection Agency (HPA). The MRL, as the national reference laboratory, obtains enhanced passive surveillance reports of malaria cases through laboratories and clinicians who are asked to provide details of the patient's age, destinations, duration and reason for the journey and use of chemoprophylaxis. The ascertainment process has been described previously and has remained unchanged over the current study period [1]. Cases were included if Sierra Leone, Nigeria, Ghana or The Gambia was said to have been visited before the onset of laboratory confirmed malaria. Malaria cases were excluded if there was incomplete information on destination or purpose of travel. Twenty nine percent of all MRL reports had missing data on reason for travel and 12% had incomplete data on the country visited [1].

### Statistical methods

The risk of malaria was estimated as the incidence rate per 1,000 person-years exposed. The numerator was the number of cases reported each year to the MRL, and the denominator was the estimated time spent in West Africa, from the IPS survey. Trend analysis was done by regression of the logarithms of the incidence rates [7]. Weights were set equal to the reciprocals of the sampling variances, which were estimated as the sum of sampling variances of the log-numerator and log-denominator. The former was estimated via the Poisson distribution as the reciprocal of the number of cases, and the latter from the 95% confidence intervals provided by the ONS. Finally, to allow for possible autocorrelation over time, the effective sample size was estimated from the regression residuals [8]. The effective sample size, which is less than or equal to the actual sample size, was used to determine the degrees of freedom for the regression analysis, which was done using the statistical package R (R Development Core Team, 2007).

## Results

### Trends in travel to West Africa

Visits by UK residents to West Africa more than doubled from 91 to 206 thousand between 1993 and 2006, an increase of 2.3-fold, or 9% per year (95% confidence interval (CI) 4.7–13%). VFR increased more than five-fold from 23 to 117 thousand, 15% per year (95% CI, 12–17%). Travel by non-VFR has risen by a third, from 68 to 89 thousand between 1993 and 2006, an increase of 4% per year (95% CI 0.5 – 8%). Since 2001, travel by VFR has been the predominant reason for visiting West Africa, with their proportion of total visits increasing from 25% in 1993 to 57% in 2006.

### Trends in travel from West Africa to the UK

**Figure 1:**
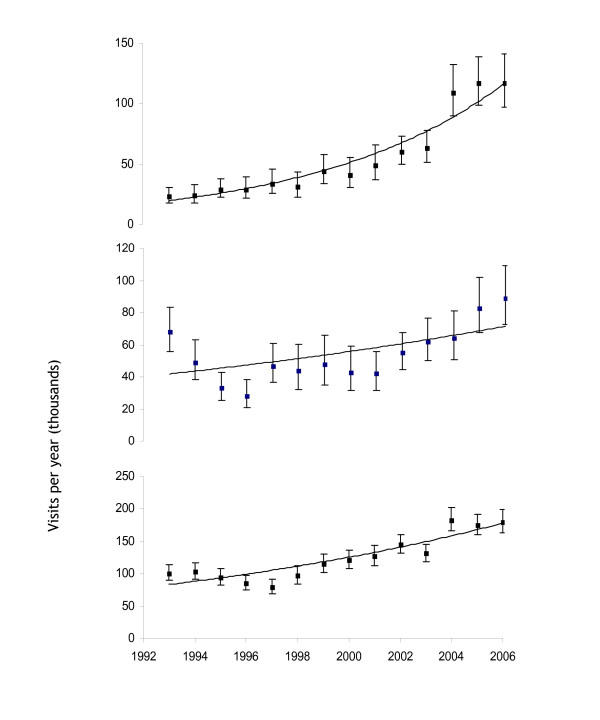
**Annual increase in visits made by the three groups of travellers to and from West Africa between 1993 and 2006.** Graphs show trend plot, mean and 95% CI. The top graph is UK residents visiting friends and relatives in West Africa, the middle graph is UK residents visiting West Africa for other reasons, the bottom graph is West African residents visiting the UK.

The number of arrivals into the UK by West African residents increased from 101 to 180 thousand between 1993 and 2006, an increase of 6% per year (95% CI 3.4 – 8.7%). Most of the arrivals were from Nigeria with just over 128,000 arrivals from this country in 2006 (Figure 1).

### Imported malaria

Of the total 8,273 malaria reports in travellers to and from West Africa during the study period, 6,655 (80%) were in UK residents visiting the region. Malaria reports per year increased by 5% from 463 in 1993 to 486 in 2006. Imported malaria cases from West Africa among VFR rose by 15% from 364 to 418 in 2006, while the number of cases in non-VFR fell by a third from 99 to 68 cases. 85% (5,676) of the malaria cases in UK residents from West Africa originated from two countries (Nigeria and Ghana), with most cases (3,820) from Nigeria, 3,402 of these cases were in VFR.

### Malaria cases in foreign visitors arriving in the UK

Of all the malaria cases occurring in visitors to the UK, one fifth (1,618 cases) were in West African residents arriving in the UK between 1993 and 2006 (range 90–154 annually). Residents arriving from Nigeria accounted for 60% (968) of all cases from the region.

### Malaria incidence rates

**Figure 2:**
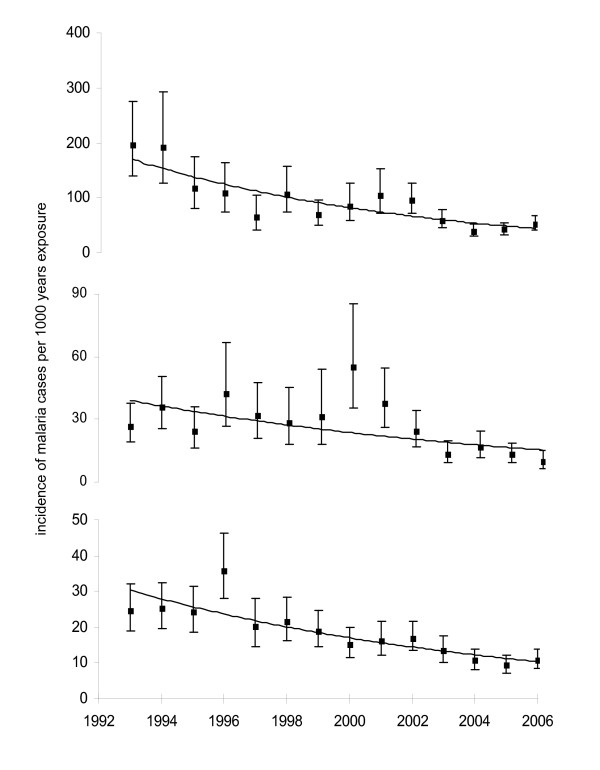
**Change in annual incidence of malaria in the three groups of travellers, graphs show trend plot, mean and 95% CI.** The slopes of the 3 curves are not signifcantly different. The top graph is UK residents visiting friends and relatives in West Africa, the middle graph is UK residents in West Africa for other reasons, the bottom graph is West African residents visiting the UK.

Linking the number of cases of malaria with the increasing time spent in West Africa shows decreasing trends in incidence rates (Figure 2). Malaria incidence in VFR reduced by a factor of 3.7 from 196 cases/1,000 person-years in 1993 to 52 cases/1,000 person-years in 2006, corresponding to an average reduction of 9.8% per year (95% CI 6.5–13%, p < 0.0001). For non-VFR the decrease was 7.0% per year (95% CI 0.4–13, p = 0.04) and for residents of West Africa it was 7.9% per year (95% CI 5.6–10%, p < 0.0001).

The non-VFR trend showed an unpredicted peak in 2000. This was the result of an increase in malaria cases occurring in tourists visiting The Gambia which apparently resulted from a weather anomaly between November and December 2000 [9].

## Discussion

This study has been possible because of the near unique denominator data collected through the International Passenger Survey which provides number of visits, reason for travel and duration of stay by UK residents by country. The IPS data, therefore, allows the calculation of rates of malaria infection over time [10]. These data have been validated against arrival statistics produced by the World Tourism Organization (WTO) [10]. Malaria notifications to the surveillance body remain considerably under-reported [11,12] and do not capture travellers who are ill abroad. However, there has been no change in reporting methodology over the study period so factors such as data quality and misclassification [13], which could affect absolute rates, would not be expected to influence the trend.

The 2.3 fold increase in travel to West Africa, and the increasing proportion of VFR, contrasts with the decade 1977–1986, when the biggest increase was due to tourism to The Gambia (528%) while all travel to Nigeria and Ghana increased by only 4% and 70% respectively [10]. Despite travel doubling over the study period, there has been almost no increase (5%) in the number of imported malaria cases. Non-VFR travellers' cases fell by a third, with VFR showing a 15% increase. Most of the malaria originated from Nigeria and Ghana, and 86% of cases were in VFR. Malaria from the Gambia is in non-VFR and remained static at around 28 cases annually until an outbreak occurred 1998–2001. The numbers of annual visits to The Gambia over this period also remained static at 37,400–40,000 per year [9]. One can only speculate what factors have led to this increase in travel to Ghana and Nigeria. The annual migration from these two countries to the UK since 1991 has doubled the numbers of settled migrants living in the UK [14,15] This expanded population would explain the increased travel by VFR to West Africa.

The proportion of UK VFR travellers to West Africa who contracted malaria was 0.5% [10] in 1986 and 1% in 1987, while in tourist and business travellers malaria occurred in 0.2% of visits in 1987 [4]. Since 1987, cases imported into the UK from all countries have remained relatively static [1], but West Africa has provided around two thirds of all falciparum malaria cases. A decline in malaria in UK travellers was first reported in 1990, where an unexplained 37% decrease in imported malaria in tourists visiting Kenya was reported [16].

Trends of imported malaria in Swedish travellers between 1997 and 2003 were studied using surveillance reports and a telephone sample survey of recent travel. Their overall rate of imported malaria from West Africa was estimated to be 30 cases per 10,000 travellers, with the risk from East African travel being less [17]. The risk of malaria from East, West and Central Africa ranged between 0.24% to 0.36% per visit. These were similar proportions to those in UK residents visiting sub-Saharan Africa over the same period: 0.14%–0.26%. The Swedish study could not separately identify VFR but the authors suggest they are a high risk group.

There are a number of factors which may have contributed to the falling incidence of malaria. The most obvious would be wider uptake of malaria chemoprophylaxis in travellers but the reverse has been reported. Studies in the late 1980s and 1990s estimated the proportion of VFR using chemoprophylaxis to be 46% [4]. A more recent analysis by Smith *et al *of malaria cases imported between 1999 and 2006 revealed that a mere 7% of VFR and 24% of non-VFR from sub-Saharan Africa used a recommended chemoprophylactic agent [1]. Krause reported that the proportion of malaria cases in Germany using any prophylaxis fell from 58% to less than 40% over the same period [18]. Nevertheless, the data are limited and some studies suffer from possible methodological problems such as possibly unrepresentative samples, so changes in the proportion of at-risk travellers using prophylaxis cannot be ruled out as an explanation of these findings.

Reduction in the intensity of malaria transmission in West Africa may have contributed to the reduced incidence in travellers. Sub-Saharan Africa has benefited from a number of campaigns to reduce the morbidity and mortality associated with the disease. The implementation of insecticide-treated bed net programmes has reduced malarial episodes in areas of stable malaria by half [19]. Reduction in malaria transmission in cities and urban environments, where many VFR visit, may also be contributing to the decline in transmission of malaria. The mean entomological inoculation rate (EIR) in urban centres compared to the mean EIR of rural areas, are 98-fold lower in dry savannas and Sahel regions, and 16-fold lower in wet savannas and forest zones [20]. Significant reductions of the burden of malaria have been reported from intervention sites in East Africa [21] following long term malaria control measures [22], including the use of artemisinin-based combination therapy and insecticide-treated nets [23]. Widespread malaria control measures may be having a similar impact on the burden of disease in travellers and West African visitors to the UK.

Accurate information on the incidence and trends of malaria in travellers has a number of obvious benefits. Policy for malaria prevention, especially chemoprophylaxis, can be more accurately targeted, when the risks are known. [24]. The future implications of this decline, should it continue, are that within the next 5–10 years the incidence could reach rates similar to those seen in South America [25] and the Indian sub-continent [24], where the risk was estimated to be < 1 case per 1000 years exposed and where the risk of adverse events are higher than the risk of acquring benign malaria. The major differences between these regions is that *Plasmodium falciparum *is the predominant species imported from West Africa, whereas *Plasmodium vivax *predominates in travellers from South America and the Indian sub-continent. Recommendations on the use of  standby treatment might provide an alternative strategy to chemoprophylaxis for travellers at such low risk of falciparum malaria and is already in use by some national bodies in low risk regions[26].

This study has been able to generate, for the first time, the incidence of malaria per unit time exposed in UK travellers to West Africa. This analysis has revealed a significant reduction in incidence of malaria over the study period in all groups of travellers but most notably in VFR. Although the reduction may have several contributory factors, the hypothesis that best links to the global reduction in incidence of travel associated malaria is reduced malaria transmission within the region. Future policy may need to accomodate the changing risk of travel associated malaria and develop new preventative strategies for low risk travellers.

## Competing interests

The authors declare that they have no competing interests.

## Authors' contributions

RHB conceived and designed the study, carried out the main analysis, data interpretation and wrote the manuscript. BC undertook the data collation, analysis and contributed to the manuscript. NA carried out the statistical analysis of the data and contributed to the interpretation of the data. VS was responsible for the collection and collation of the malaria surveillance data. All authors provided critical reviews of the manuscript and approved the final version.
